# MDR Tuberculosis Treatment

**DOI:** 10.3390/medicina58020188

**Published:** 2022-01-26

**Authors:** Juan Espinosa-Pereiro, Adrian Sánchez-Montalvá, Maria Luisa Aznar, Maria Espiau

**Affiliations:** 1 Infectious Diseases Department, Vall d’Hebron University Hospital, PROSICS Barcelona, Universitat Autònoma de Barcelona, 08135 Barcelona, Spain; jespinosa@vhebron.net (J.E.-P.); adsanchez@vhebron.net (A.S.-M.); 2 Mycobacteria Infection Study Group from Spanish Society of Infectious Diseases and Clinical Microbiology, 28003 Madrid, Spain; 3 Pediatric Infectious Diseases and Immunodeficiencies Unit, Vall d’Hebron University Hospital, Universitat Autònoma de Barcelona, 08135 Barcelona, Spain; mespiau@vhebron.net

**Keywords:** tuberculosis, MDR, treatment

## Abstract

Multidrug-resistant (MDR) tuberculosis (TB), resistant to isoniazid and rifampicin, continues to be one of the most important threats to controlling the TB epidemic. Over the last few years, there have been promising pharmacological advances in the paradigm of MDR TB treatment: new and repurposed drugs have shown excellent bactericidal and sterilizing activity against *Mycobacterium tuberculosis* and several all-oral short regimens to treat MDR TB have shown promising results. The purpose of this comprehensive review is to summarize the most important drugs currently used to treat MDR TB, the recommended regimens to treat MDR TB, and we also summarize new insights into the treatment of patients with MDR TB.

## 1. Introduction

Tuberculosis (TB) continues to be one of the 10 main causes of death in the world, being, since 2015 and until the COVID-19 pandemic, the leading cause of death from a single infectious agent (ranking above HIV/AIDS), responsible for 1.3 million deaths among people not infected with human immunodeficiency virus (HIV) and an additional 214,000 deaths among HIV-infected people [1]. Although there has been a decrease in TB incidence and mortality in recent years, we are still far from the global TB targets proposed by the WHO in its “End TB” strategy launched in 2015 that aims to reduce the absolute number of TB deaths by 95% and the absolute number of new cases TB by 90% by 2035 [2]. The appearance of *Mycobacterium tuberculosis* (MTB) strains resistant to the most effective drugs is one of the main problems that contribute to the slow decline in TB cases. Multidrug-resistant TB (MDR TB), defined as TB caused by MTB bacilli resistant to rifampicin (RIF) and isoniazid (INH), represents a major threat to global TB control. From a surveillance perspective, data on MDR TB are aggregated with RIF resistant TB (RR-TB), since many settings rely on molecular methods to test RIF sensitivity, which is a subrogate marker of resistance to RIF and INH. Globally, in 2020, 132,222 cases of MDR/RR-TB were documented, and only one out of three cases were enrolled into an adequate treatment program, and only 59% of those treatments were completed. However, the relapse rate in real-world practice is commonly missing, making it difficult to calculate the size of the problem [1]. Hence, there are still important barriers regarding access to diagnostic tests and adequate treatment.

Until 2016, MDR TB treatment was based on an injectable drug plus a fluoroquinolone (moxifloxacin, levofloxacin, or gatifloxacin) with a duration of 18–20 months or longer [3]. In 2016, the WHO treatment guidelines for MDR TB recommended for the first time the use of a short treatment regimen in selected patients, based on the results of several published studies that included cohorts of MDR TB patients treated with regimens with a duration between 9 and 12 months [4,5,6,7]. Despite the incorporation of these abbreviated schemes, the overall success of MDR TB treatment remained far from the 2035 milestones [2]. Moreover, the treatment regimen includes supplementation with an injectable drug and high-dose INH, responsible for a high proportion of side effects including hearing and renal impairment.

In recent years, three new drugs for the treatment of TB have emerged: bedaquiline (BDQ), delamanid (DLM), and pretomanid (PTM), which have been shown to improve the percentage of therapeutic success in patients with MDR TB [8,9,10]. In addition, repurposed drugs, such as linezolid (LZD) and clofazimine (CFZ), have strengthened the evidence of alternative drugs for the treatment of MDR TB [11,12,13,14]. Several studies have assessed an all-oral (without using an injectable aminoglycoside) shorter new regimen with promising results. As a consequence, new WHO guidelines for MDR TB treatment have substantially changed, recommending shorter all-oral treatment for MDR TB [15]. Unfortunately, all-oral shorter regimen recommendations still advocate for 9–12 month treatment duration with a combination of seven drugs including, among others, BDQ, levofloxacin/moxifloxacin and high-dose INH. DLM and LZD, despite good results in real-world data, they are not yet included in the recommendations for shorter treatment due to the insufficient quality of the data [16]. These changes in MDR TB treatment recommendation have led to updates in the drug classification for MDR TB, and in the definition of extremely resistant TB (XDR TB) and pre-XDR TB. XDR TB is now defined as TB caused by MTB strains that fulfil the definition of MDR/RR-TB and are additionally resistant to any fluoroquinolone and at least one additional Group A drug (BDQ and LZD). Pre-XDR TB is defined by a TB caused by an MTB strain that fulfils the definition of MDR/RR-TB and is also resistant to any fluoroquinolone [17].

In this review, we intend to summarize the main aspects of new and repurposed drugs used for the treatment of MDR TB. We designed the manuscript to help clinical doctors better understand the pharmacologic characteristics as well as to give insight into how to use the individual drugs while performing an adequate follow up and adverse effect management. We believe that patients with MDR TB should be managed by expert personnel, and tailored treatments should be considered to address patients’ needs and preferences. We have added a section on TB in children concerning each drug to revise the available evidence in this subgroup of vulnerable patients. 

## 2. MDR TB Recommended Regimens and Duration

The following regimens are currently recommended by the MDR TB WHO guidelines. These regimens are summarized in Table 1.

- A 9–12 month duration of BDQ (used for 6 months), levofloxacin/moxifloxacin, ethionamide, ethambutol, INH (high-dose), pyrazinamide, and CFZ for 4 months (with the possibility of extending to 6 months if the patient remains sputum smear positive at the end of 4 months), followed by 5 months of treatment with levofloxacin/moxifloxacin, CFZ, ethambutol, and pyrazinamide. This regimen is recommended for patients who have not been exposed to treatment with second-line TB medicines for more than 1 month and in whom resistance to fluoroquinolones has been excluded [18]
- A regimen lasting 18–20 months composed of all three Group A agents (i.e., BDQ, levofloxacin/moxifloxacin, and LZD) and at least one Group B agent (i.e., cycloserine and CFZ) to ensure at least four TB agents that are likely to be effective and that at least three agents are included for the rest of the treatment if BDQ is stopped. Both Group B agents are to be included if only one or two Group A agents are used. If the regimen cannot be composed of agents from Groups A and B alone, Group C (i.e., ethambutol, DLM, pyrazinamide, imipenem–cilastatin or meropenem, amikacin (or streptomycin), ethionamide or prothionamide, and p-aminosalicylic acid) agents are added to complete it. In children, the duration of therapy depends on the site and severity of the disease: non-severe disease can be treated for 9–12 months, while severe disease will require 12–18 months of therapy [19]. Vitamin B6 must be given to all children receiving therapy for MDR TB [19]
- A 6–9 month regimen of BDQ, PTM, and LZD under operational research in patients with resistance to fluoroquinolones who have not received previous treatment with BDQ and LZD for more than 2 weeks [20].

**Table 1:** Current recommended treatments for MDR and XDR TB (2020 WHO guidelines).

Regimen	Composition	Total Duration	Observations
Long MDR TB	6 months > 4 drugs (3 group A + 1 − 2 group B) 12–14 months and 3 drugs (BDQ/AMK/DLM stop) **	18–20 *	At least 4 effective drugs at the beginning; LZD at least 6 months
Short MDR TB	4–6 months > Km ^†^ + MFX + CFZ + ETO + Z + E + High-Dose INH 5 months > MFX + CFZ + Z + E	9–12	Exclusion: >1 previous month on any of these drugs, extrapulmonary TB in persons living with HIV, miliary TB or TB meningitis
	4–6 months > BDQ + MFX + CFZ + ETO + Z + E + High-Dose INH 5 months > MFX + CFZ + Z + E	9–12	Exclusion: Extensive pulmonary TB, miliary or TB meningitis, resistance to fluoroquinolones
	BDQ + PTM + LZD	6–9	XDR or MDR with no alternative regimen

BDQ: bedaquiline, AMK: amikacin, DLM: delamanid, Km: kanamycin, Mfx: moxifloxacin, CFZ: clofazimine, ETO: ethionamid, Z: pyraizinamid, E: ethambutol, INH: isonizid, TB: tuberculosis, HIV: human immunodeficiency virus, LZD: linezolid. * Children with non-severe disease can be treated for 9–12 months, while children with severe disease will require 12–18 months ** BDQ and DLM may be considered for use longer than 6 months. ^†^ Kanamycin in STREAM trials. However, considering later evidence, the guidelines recommend using amikacin.

## 3. Main Drugs Used to Treat MDR TB

In this section, the principal drugs used to treat MDR TB are described. Table 2 summarizes the main characteristics of new and repurposed drugs used to treat MDR TB. 

### 3.1. Diarylquinolines: Bedaquiline (BDQ)

#### 3.1.1. Mechanism of Action

BDQ is the first drug with a novel mechanism of action against MTB to receive FDA accelerated approval in 40 years [21]. It is a diarylquinoline that inhibits ATP synthase, and is bactericidal to actively replicating and non-replicating mycobacteria [22,23,24].

#### 3.1.2. Mechanism of Resistance

The main mechanisms that confer resistance to BDQ are:

1. Mutations in the *atpE* gene, which encodes the ATP synthase. This mutation alters the union between ATP synthase and BDQ, and it has been linked to resistance both in vitro and in vivo [25,26];
2. Mutations in the *Rv0678* gene that codes for a drug efflux pump regulator. This mutation has been postulated to confer “low-level” resistance to both BDQ and CFZ, and the impact of this mutation on treatment outcomes must be determined [27,28];
3. Mutations in the gene *pepQ* (Rv2535) confer BDQ and CFZ low-level resistance. The mechanism behind the resistance remains unclear [29].

Acquired resistance to BDQ during MDR TB treatment have been reported and underlies the need of using proper accompanying anti-TB drugs to which the patient’s isolate has shown to be susceptible. Laboratory surveillance systems are of the utmost important for early detection of the emergence of resistance under programmatic conditions and should be strengthened after the introduction of a new drug [30]. Importantly, based on the resistance mechanism, cross-resistance to CFZ must be considered when designing the MDR TB regimen [31]. Moreover, due to the long half-life of BDQ, it is possible that patients who have not culture converted when the BDQ-containing regimen was discontinued may select for BDQ resistance. 

#### 3.1.3. Posology

The recommended dose of BDQ for the treatment of pulmonary MDR TB in adults is 400 mg administered orally once daily for 2 weeks, followed by 200 mg administered orally three times weekly. In patients weighing <30 kg, the recommended dose is 200 mg once daily for 2 weeks, followed by 100 mg once daily three times a week [16]. Additionally, alternative daily posology (200 mg daily for 8 weeks, followed by 100 mg daily) is being explored in at least two clinical trials (Zenix (NCT03086486) and SimpiciTB (NCT03338621)) based on pharmacokinetic simulations that provide similar drug levels compared to the approved posology. BDQ shows better absorption when the drug is taken with food [32]. The WHO recommends using BDQ for a maximum of 24 weeks. However, recent studies have reported the safeness of using BDQ for longer periods of time, without having observed relevant adverse events [33,34].

#### 3.1.4. Efficacy

Efficacy of BDQ in patients with pulmonary MDR TB was first demonstrated in 2014, when the addition of BDQ to a background regimen showed a faster and increased number of culture conversions at 120 weeks compared with the placebo (79% *vs.* 58%) [9]. In an individual patient data meta-analysis, where 12,030 patients from 50 studies were included, both treatment success and mortality reduction were associated with the use of BDQ [35]. Recently, the NIX-TB study, which evaluated the safety and efficacy of a regimen containing BDQ, PTM, and LZD for 26 weeks in patients with XDR TB and patients with MDR TB that discontinued the second-line regimen, demonstrated that 90% of patients had a favorable outcome after 6 months of treatment [20]. Moreover, in the PRACTECAL study, a regimen of BDQ, PTM, LZD, and moxifloxacin over 6 months was observed to be superior to the standard of care in patients with MDR TB with 89% of patients achieving a successful treatment (congress communication) [36].

#### 3.1.5. Adverse Events

BDQ is one of the drugs used for MDR TB with a better safety profile, showing a low incidence of adverse events leading to permanent drug discontinuation [37]. 

The most common adverse events related with BDQ are headache, nausea, vomiting, and arthralgia. Elevation of liver enzymes has also been reported. However, the most concerning adverse event is QTc prolongation, which is also related with other drugs used to treat MDR TB such as fluoroquinolones, DLM, and CFZ [38,39]. So, when BDQ is included in the regimen, it is recommended to obtain ECGs after the initial 2 weeks of therapy and then at monthly intervals to monitor for QTc prolongation. Serum electrolytes should also be monitored. If there are other conditions that increase the risk of QTc prolongation, weekly ECG should be recommended [40]. 

#### 3.1.6. Interactions

BDQ is metabolized in the liver by cytochrome P450 (CYP) isoenzyme 3A4. Consequently, drugs that inhibit cytochrome P450 3A4 (CYP3A4) could result in increased concentrations of BDQ, which could increase toxicity, whereas drugs that induce CYP3A4 activity could result in reduced concentrations of BDQ [32]. Among all possible interactions, the potential one between BDQ and antiretroviral drugs should be noted. HIV protease inhibitors may increase BDQ concentrations, whereas BDQ may reduce the concentrations of lopinavir (LPV) [41]. It has also been observed that efavirenz may decrease the concentrations of BDQ, so the use of efavirenz and protease inhibitors should be avoided in patients receiving BDQ [42]. The WHO recommends the use of two nucleoside reverse transcriptase inhibitors (NRTIs) with nevirapine or triple NRTI to treat HIV in patients receiving BDQ containing regimens. However, there is concern whether switching efavirenz-containing regimen to nevirapine-containing regimen may reduce antiretroviral efficacy and increase the risk of viral failure and emergence of resistance [43]. Theoretically, integrase inhibitors not boosted with cobicistat may stand as an alternative when considering the use of BDQ. 

#### 3.1.7. Children

BDQ may be used as part of the shorter all-oral BDQ-containing regimen (conditionally recommended by WHO in 2020) or as part of longer treatment regimens in children of all ages (previously, only in patients aged > 5 years). This recent change in the recommendations of the WHO was based on preliminary data from two phase II trials: TMC207-C21112 (NCT02354014) and IMPAACT P110813 (NCT02906007) that reported no cardiac safety concerns from those reported in adults. Population pharmacokinetics (PK) models from both studies suggest that drug levels observed in adults can be reached in most children receiving BDQ, although some dose modification may be necessary depending on the age and weight of the child [44].

The recommended dosage is 6 mg/kg/day for 14 days, then 3–4 mg/kg three times/week for 22 weeks [45]. Treatment with BDQ is usually for six months, but there are no known safety issues when this drug is used for longer than six months (although data are limited). Like adult patients, children may benefit from using BDQ for the full duration of their therapy [18].

If cardiac toxicity occurs (clinically significant ventricular arrhythmia or QTc interval >500 ms), therapy should be discontinued. Other possible adverse events include neurological toxicities such as paresthesia, tremor, anxiety, depression, insomnia, tinnitus, and blurred vision. In case of severe renal and/or hepatic impairment at initiation, close monitoring is advised. In these situations, dose adjustment is not recommended according with the manufacturer’s labeling, although caution should be observed. If hepatotoxicity occurs during therapy, therapy should be discontinued if any of the following presents: aminotransferase elevation and total bilirubin >2 times upper limit of normal (ULN), aminotransferase elevation >8 times ULN, or aminotransferase elevation >5 times ULN.

### 3.2. Nitroimidazoles: Delamanid (DLM) and Pretonamid (PTM)

Two nitroimidazoles, DLM and PTM, have shown to be active against MTB, and are included in the WHO guidelines to treat MDR TB. DLM obtained FDA conditional approval in 2014, while PTM was approved under the Limited Population Pathway for Antibacterial and Antifungal Drugs in 2019. 

#### 3.2.1. Mechanism of Action

Both nitroimidazoles impair the biosynthesis of methoxy- and keto-mycolic acids, which are components of the mycobacterial cell wall [46]. Both compounds are prodrugs that are activated by the MTB-reductive metabolism to produce an active free radical via the mycobacterial F420-dependent reductase coenzyme system [47].

In anaerobic or hypoxic conditions, these drugs act against non-replicating bacilli [48,49].

#### 3.2.2. Resistances

DLM and PTM are prodrugs that require metabolic activation involving coenzyme F420. Mutations in the genes implicated in prodrug activation (*fgd1* and *ddn*) and F420 biosynthetic pathway (fbiA, fbiB, fbiC) have been described as resistance mutations [47,50,51]. The emergence of DLM resistance has been observed during MDR TB treatment [52,53]. Phenotypic resistance to DLM has been observed in patients with MDR TB who have not been previously treated with DLM. The proportion of pre-existing DLM resistance widely depend on the minimum inhibitory concentration (MIC) threshold used, with higher proportion reaching 9.76% in Korea when the limit was set at 0.2 mg/dL as suggested by the manufacturer [54].

#### 3.2.3. Posology

DLM is available as tablets (50 mg) and the recommended dose for patients weighing 50 kg and above is two tablets twice a day taken with food. Patients weighing between 30 and 50 kg should take one tablet twice a day [55]. As per BDQ, WHO recommends using DLM for a maximum of 24 weeks. However, experiences with the use of this drug beyond 24 weeks have not shown relevant adverse events [33,34].

WHO guidelines recommend using PTM at 200 mg once daily, as per the Nix-TB study regimen [20]. Co-administration with food results in higher levels of PTM. There is no data for extended use of PTM beyond 6 months.

#### 3.2.4. Efficacy

The efficacy of DLM was first observed in 2012, when the supplementation of a background drug regimen with DLM 100 mg twice daily resulted in an increase in sputum-culture conversion at 2 months among patients with MDR TB in a phase II trial [10]. In 2013, in the open label extension trial, patients in the under DLM treatment resulted in improved success outcomes and reduced mortality [56]. However, other studies reported conflicting results. In a randomized, double-blind, placebo-controlled phase 3 clinical trial conducted by Otsuka pharmaceutical, patients with pulmonary MDR TB were randomized to receive supplementation with DLM plus optimized background regimen based on WHO guidelines. Primary outcomes were time to sputum culture conversion at 6 months post-randomization in the intention to treat population. Median time to sputum culture conversion was 51 days (29–98) for DLM vs. 57 days (43–85) for the placebo arm (HR 1.17 (95% CI 0.91–1.51) [57]. In a prospective study comparing the outcomes of patients with MDR TB receiving a BDQ-based *vs.* DLM-based regimen, sputum culture conversion and favorable clinical outcomes were higher in patients receiving BDQ versus DLM-based regimens [58]. In contrast, differences in the final treatment outcome between patients with MDR/RR-TB who received BDQ-based regimens and those who received DLM-based regimen were not observed in the other cohort of patients [59]. Waiting for new results, DLM is classified as group C in the WHO grouping of medicines recommended for use in longer MDR TB regimens [15].

In 2015, the use of PTM was studied during an 8 week phase 2b study in combination with moxifloxacin and pyrazinamide in both RIF susceptible (Rs TB) and RR-TB. This regimen was observed to be well tolerated and showed superior bactericidal activity in drug-susceptible TB than the current standard of care. In MDR TB, the regimen was not compared to others, but it was comparable to the standard treatment for Rs TB (INH, RIF, pyrazinamide, and ethambutol; HRZE) in Rs TB [60]. In 2019, a regimen containing moxifloxacin, BDQ, PTM, and pyrazinamide assessed the response in patients with RR-TB over 8 weeks. This regimen showed a faster culture conversion compared to Rs TB patients on HRZE [61].

PTM received approval in 2019 by the FDA for treatment of pulmonary XDR TB and non-responsive MDR TB due to the recent results of the Nix-TB study in which a treatment regimen containing BDQ, PTM, and LZD for 6 months observed high percentages of favorable outcomes among patients with MDR and XDR TB [20].

DLM has greater in vitro potency against MDR TB and XDR TB isolates than PTM [62,63].

#### 3.2.5. Adverse Events

Most common adverse events related to both DLM and PTM are gastrointestinal. Both DLM and PTM may cause QTc prolongation, which is an adverse event associated with other drugs used to treat MDR TB such as BDQ and fluoroquinolones. However, updated evidence did not report an increase in clinically significant cardiac adverse events when combining DLM with other drugs with potential to prolong QTc [64]. 

Liver-enzyme increases have also been observed during treatment with DLM- and PTM-containing regimens without major consequences when managed properly [65].

#### 3.2.6. Interactions

DLM does not interact with the CYP enzymes, so it is unlikely to cause clinically relevant drug–drug interactions when co-administered with products that are metabolized by the CYP enzyme system [66]. There are no clinically significant interactions when DLM is co-administered with efavirenz, LPV/ritonavir (LPV/r), or tenofovir [55].

In a Phase I study regarding pharmacokinetics of PTM, concomitant use of LPV/r only modestly reduced PA-824 plasma levels, suggesting that the drugs can be co-administered without dose adjustment. Efavirenz reduced PTM concentrations more substantially, and RIF reduced PTM concentrations even more [67]. The clinical significance of this interaction is unknown, although precaution should prevail if co-administered.

#### 3.2.7. Children

Since 2018, WHO has conditionally recommended the use of DLM for the treatment of MDR/RR-TB patients aged 3 years or more for longer regimens, based on extrapolation of efficacy data in adults, and trial data on pharmacokinetics and safety in children [15]. 

However, recommendations were recently extended to children of all ages after reviewing preliminary data from a phase I, open-label, age de-escalation trial designed to assess the pharmacokinetics, safety, and tolerability of DLM administered twice daily for 10 days in children with MDR/RR-TB and from the corresponding open-label extension study (NCT01856634). Drug concentrations in the 0–2-year age group were lower than those of patients aged 3 years and older, necessitating a modeling/simulation approach to dosing. No cardiac safety signals distinct from those reported in adults were observed in children 0–2 years of age. Pharmacodynamic simulations suggested that clinically meaningful changes in QTc interval would be unlikely in children under 3 years of age, even if higher doses were used to reach drug concentrations comparable to those achieved in adults [44].

Experts recommend that DLM should replace injectable agents in children with MDR/RR TB at a daily dose of 3–4 mg/kg (to a maximum of 200 mg) [45]. The WHO recommends a dosing approach depending on age: 25 mg orally twice daily in 3–5 years (7–23 kg); 50 mg orally twice daily in 6–11 year olds (24–4 kg); 100 mg orally twice daily in 12–17 year olds (>34 kg) (upper daily dose 200 mg) (WHO has not yet made an official recommendation on the appropriate dosage in children under 3 years of age) [15]. Same as with BDQ, licensing for DLM is usually for six months, but there are no known safety concerns when using this drug for longer than six months (although data are limited). Some children may benefit from longer treatment periods [18].

Preliminary pediatric data from Otsuka revealed a significant temporal trend with QTc increasing over the first month of DLM administration before plateauing and returning to baseline. Prolonged QTc is exacerbated by hypoalbuminemia, hypokalemia, and other QTc-prolonging medications. To date, DLM has not been associated with any severe adverse events in children, but neurologic toxicities, such as paresthesia, tremor, anxiety, depression, insomnia, tinnitus, and blurred vision, can occur [45]. 

The safety of PTM in infants and children has not been adequately evaluated. Until more data are available, DLM should be prioritized when designing an MDR TB regimen [15]. 

### 3.3. Oxazolidinones: Linezolid (LZD) and Tedizolid (TDZ)

Oxazolidinones are a class of synthetic antibacterial agents that were first produced in 1978 for agriculture. In the 1980s, the first attempts on human use started [68].

However, their development was hindered by unacceptable toxicity until LZD became available one decade later [69]. LZD was approved by the FDA in 2000 for the treatment of Gram-positive infections, but its use in TB is still off-label. Conditional approval was expedited by FDA for the use of LZD in combination with BDQ and PTM for non-responsive MDR TB or XDR TB after the results of the Nix-TB trial [20]. TDZ is another approved oxazolidinones with similar indications than LZD. Experience of TDZ is very limited due to the costs. Despite this, there are limited data on prolonged exposure to TDZ, and the safety profile seems to be better than that of LZD. TDZ use in TB is off-label.

#### 3.3.1. Mechanism of Action

Their spectrum includes Gram-positive bacteria (*staphylococci, streptococci*, *enterococci,* and *Bacteroides fragilis*, among others) and mycobacteria (MTB and *M. avium complex*) [70]. Oxazolidinones inhibit the protein synthesis by blocking the formation of the initiation complex as they bind to the 50S ribosomal subunit near to the interface with the 30S subunit [69]. Like other antibiotics inhibiting protein synthesis, the oxazolidinones may hinder the production of protein virulence factors [71]. 

TDZ appears to be 4–16-fold more potent in vitro than LZD. When used at standard doses, the drug concentrations of LZD are at least twice that of TDZ, resulting in area under the curve (AUC)/CMI ratios that are similar. The main advantage is therefore that similar efficacy may be achieved with less drug concentrations and thus less risk of toxicity [72].

#### 3.3.2. Mechanism of Resistance

Resistance develops by target mutations that modify the ribosomal subunit at the drug-binding domain [73]. Although several other antibiotics inhibit protein synthesis, no cross-resistance has been reported. It has been hypothesized that this lack of cross-resistance depends on two facts: (1) the drug-binding domain is far away from that of other antibiotics inhibiting drug synthesis; (2) the initiation process that the oxazolidinones inhibit takes place prior to that of other protein synthesis inhibitors that prevent the elongation process [74]. 

TDZ may preserve its activity in Gram-positive bacteria in the presence of LZD resistance mutations, but it is not the same the other way around [75]. TDZ resistance can appear in Gram-positive bacteria when plasmid mutated *cfr* occurs at the same time with chromosomal resistance to LZD [76].

#### 3.3.3. Posology

Oral bioavailability of LZD is close to 100% [77]. Additionally, the presence of food or the co-administration of aluminum-based antacids or proton-pump inhibitors does not seem to affect LZD absorption [78,79]. Thus, drug concentrations with oral and intravenous administration can be considered equivalent in stable patients. LZD has a large volume of distribution, and its clearance is linear at therapeutic doses. Importantly, no differences in drug concentrations were shown in elderly persons or in those with mild-to-moderate renal or liver function impairment. Drug concentrations increase in persons who need hemodialysis, and it decreases in children [80]. In the treatment of MDR TB, LZD can be used orally or via intravenous infusion at a dose of 600 mg twice a day, in combination with other drugs [15,35]. One recent study used 1200 mg once daily in combination with BDQ and PTM [20]. In our experience, LZD is well tolerated at the usual dose of 600 mg twice daily for the first weeks. When the patient status improves, the dose can be adjusted to 600 mg once daily to avoid adverse events. Reduction to 300 mg daily has also been implemented successfully in combination with other active drugs [81]. However, when possible, the maximum tolerable dose should be given with an optimal dose of ≥600 mg daily, since a daily dose of 300 mg may not be optimal for patients with a high bacillary load or infections with MTB with a MIC to LZD superior to 0.125 mg/L [82]. We recommend performing target drug monitoring to assess efficacity and to reduce the probability of side effect by achieving a minimum trough concentration inferior to 2 mg/L. For those receiving intermittent hemodialysis, the dose should be administered after each session.

There is less evidence for the optimal posology of TDZ in MDR TB. The FDA-approved dose for skin and soft tissue infections is 200 mg once daily, either oral or intravenous. Its bioavailability is approximately 91%, and most of the drug is protein-bound in the blood, and its main elimination route is with the feces.

One study evaluated the weekly administration of TDZ in a hollow-fiber model of TB, showing that this drug can have a good sterilizing effect even with intermittent administration. In that study, Monte Carlo simulations showed that 200 mg daily, 700 mg twice a week, or 1400 mg once a week could be explored for their sterilizing effect in human trials [83]. 

#### 3.3.4. Efficacy

Although the protein synthesis inhibiting antibiotics are generally considered to be bacteriostatic, LZD has been shown to have potent activity against MTB both in vitro and in vivo [12,84]. In addition, there seems to be a synergistic effect when combined with moxifloxacin [85]. When the dose is lowered to 600 mg per day, the AUC/MIC ratio remains above the target for MTB with MIC for LZD of 0.25 mg/L [86], and its anti-TB efficacy is not affected in vivo for patients with strains with MICs below the mentioned threshold [87]. Doses of 300 mg have also proven to be effective in patients with MDR TB, with a very good safety profile. However, LZD exposure decreases and careful interpretation of the MIC to LZD is mandatory, and target drug monitoring is highly recommended [12]. 

Regarding clinical results MDR TB, a meta-analysis including 12 randomized trials showed that more than 90% of the participants receiving a LZD containing regimen converted sputum smear or culture after a median of 43.5 (IQR 21–90) and 61 (29–119) days, respectively. Treatment failure and death were observed in 4.1% and 14.1%, respectively [11]. Consistently, a recent patient-level meta-analysis including 87 studies, including 799 who received LZD and 5864 who did not, showed a benefit of LZD in treatment success (adjusted OR 3.4, 95% CI 2.6–4.5) and death (adjusted OR 0.3, 95% CI 0.2–0.3) [35]. Another meta-analysis focused on complicated MDR TB (defined as those cases previously treated with second-line drugs fulfilling the formed definition of XDR-TB) showed that 68% of the 148 participants from 11 studies had treatment success. Among those who suffered a poor outcome, 18 died, 11 had treatment failure, 10 defaulted, and two had no further information [88]. All these data, together with information from Nix-TB study place LZD on the front-line of drugs for the treatment of MDR TB. The optimal dose needs to be defined, dose adjustment based on strain susceptibility, and patient exposure are recommended [20]. From a programmatic perspective, a 1200 mg daily dose of LZD at the first weeks of the treatment with a reduction to 600 mg daily when conversion to smear-negative or if appearance of adverse effects may offer good results.

TDZ has the advantage of a higher concentration in the epithelial-lining fluid relative to plasma concentration when compared with LZD. It is still unknown its penetration into the cavern or the caseum material [89]. TDZ has good in vitro activity against susceptible and MDR TB and good intracellular killing activity comparable to that of RIF [90,91]. Hollow-fiber models show that the combination of TDZ with moxifloxacin and faropenem could have faster sterilizing activity compared with RIF–INH–pyrazinamide for non-replicating bacteria [92]. In murine models, TDZ combined with BDQ and PTM had sterilizing activity superior to the standard regimen. TDZ has shown it is well tolerated with low adverse effects in short-term and long-term uses, and it appears to have a more favorable adverse effects profile than LZD [93]. 

As the pivotal studies for TDZ approval included complicated skin and soft tissue infections, the information about its efficacy in TB is very scarce. One case report showed culture conversion after 1 month with TDZ 200 mg daily plus levofloxacin 750 mg daily, ethambutol 25 mg/kg/day, and nebulized amikacin 500 mg twice daily. The patient was an adolescent that needed a liver transplant after first line anti-TB drugs induced liver failure [94]. The optimal combination regimen for TDZ is still not known. Based on in vitro and preliminary in vivo data, it could be used as an alternative to LZD with a better safety profile, although more data from clinical trials are needed.

#### 3.3.5. Adverse Events

In general, the most common reactions in the short term are gastrointestinal effects (diarrhea and nausea), headache, and rash [69]. Severe adverse events leading to interruption of treatment with LZD happen in about 3–4% of the patients with short course treatment [69]. One of the most worrisome adverse effects of LZD is myelosuppression, affecting about 28–33% of the patients using LZD for long periods [88,95]. Early reports showed that this toxicity is dose-dependent and tends to appear after at least 2 weeks of treatment. Therefore, myelotoxicity is less probable in patient under doses of ≤600 mg daily, even in treatments longer than 20 months [96]. Bone marrow recovers within one to three weeks after LZD withdrawal [97,98]. Therefore, hematologic monitoring is recommended in patients undergoing treatment with regimens containing LZD for longer than one week [97,98]. The most frequent hematologic alteration is thrombocytopenia followed by anemia [99]. Some authors recommend supplementation with iron and folic acid during LZD treatment to reduce the risk of myelotoxicity [96]. There is no “acceptable” range of hematologic toxicity. As the condition of TB patients is often frail, some degree of anemia and thrombocytopenia are to be expected during the first weeks of treatment, but their progression needs careful assessment, especially in those patients with added risks for myelosuppression (advanced age, concomitant drugs such as cotrimoxazole, and hematological diseases). 

Neurologic toxicity mainly manifested as peripheral and optic neuropathy. It can affect around 30–36% of the patients under long-course treatments [88,95]. This toxicity is also time- and dose-dependent, appearing after a median of 5–11 months with the 600 mg twice-daily dose [100,101]. It has been suggested that this effect depends on the toxic effects on mitochondria [97,102,103]. Peripheral neuropathy often presents with paresthesia, burning pain, and hypoesthesia with a “glove and stocking” distribution [100,101]. Optic neuropathy presents with visual impairment, scotomas, and color-perception impairment [97]. Optic neuropathy may be reversible, but peripheral neuropathy can be irreversible in some cases with evidence of axonal damage in the electromyogram showing mixed axonal damage [97,104,105]. Those receiving treatments for more than one month should be carefully evaluated for early signs or symptoms of optic and peripheral neuropathy, and the treatment stopped should any of these arise [106]. When possible, therapeutic drug monitoring can help adjust the LZD dose, a trough concentration < 2 mg/L to minimize LZD toxicity. 

In the pivotal studies ESTABLISH-1 and -2, fewer participants discontinued the assigned regimen in the TDZ arm than in the LZD arm due to adverse events. For both arms, the most frequent adverse events were gastrointestinal [107,108,109]. Interestingly, there were eight (1.2%) cases of peripheral neuropathy in the TDZ arms and four (0.6%) in the LZD arms [110]. TDZ toxicity beyond the first weeks of treatment was scarce. In a case report of an adult with recurrent methicillin-resistant *S. aureus* infections who received TDZ for 18 months, evidence of hematological or neurological toxicity was not observed [111]. In the case report of a liver-transplant recipient patient with pulmonary drug susceptible TB (Ds-TB), no hematologic toxicity was noted during the 20 month treatment [94]. Finally, in a series of 25 patients treated with TDZ for non-tuberculous mycobacteria for a median of 14 weeks, five (21%) had peripheral neuropathy, one lymphopenia, one thrombocytopenia, and one anemia [112]. 

#### 3.3.6. Interactions

LZD acts as a monoamine oxidase inhibitor (MAOI) and, therefore, can trigger a serotonin syndrome when co-administered with bupropion and other drugs that act as MAOI or inhibit serotonin reuptake (see Appendix A) [113,114]. It must be noted that these drugs are common among the chronic treatments of aged patients and patients with psychiatric diseases. Similarly, tyramine-rich foods can cause hypertensive crises or serotonin syndrome [115]. Opioids drugs have the potential to interact with MAOI, although the degree of the interaction vary according with the type of opioid drug. The phenylpiperidine opioids (i.e., pethidine, tramadol, methadone, and fentanyl), and dextromethorphan and propoxyphene have been implicated in a number of case reports of serotonin toxicity [116]. Serotonin syndrome typically manifests as fever, rash, agitation with change in mental status, and tremors, and it can be life-threatening in some cases [117]. Regarding interactions with other antibiotics that may be used against mycobacteria, RIF reduces the concentration–time curve and the maximum concentration of LZD in about 32% and 21%, respectively [118]. Clarithromycin appears to increase LZD plasma concentration [119].

TDZ is also a nonselective MAOI, but it did not show a clinically evident effect in murine model [72]. However, as said above, the pivotal trials used TDZ for very short periods, and excluded participants taking drugs that could interact with TDZ and/or increase serotonin levels [107,108]. Therefore, there is not enough evidence to rule out serotonin syndrome when co-administered with other drugs that increase plasma serotonin levels. 

#### 3.3.7. Children

The WHO recommends that LZD is included as a core agent in the treatment of children with MDR TB, using a regimen with at least four active agents [15]. 

Dosages of 10–12 mg/kg once daily for children who weigh ≥16 kg (not exceeding 600 mg daily) and 15 mg/kg once daily for children weighing <16 kg are recommended in patients under 15 years (orally or IV). For children 15 years old or older, adult recommendations apply [15]. Potential twice daily dosing in children with extensive disease or TB meningitis can be considered, at least initially. Use throughout treatment is likely to improve efficacy, although adverse events may limit the duration of use to the first few months. So, the treatment duration is dependent upon clinical course and tolerance; a range of 13–36 months in pediatric patients has been described [18].

Experience in pediatric patients reflects extrapolation of dosing approach used in adult patients which includes a lower daily dose to decrease risk of adverse effects due to the anticipated long duration of therapy and, if toxicity does occur, further dosage reductions (a 25–50% dose decrease or increased dosing interval have been used) [45]. 

Toxicity is time- and dose-dependent, bone marrow suppression and peripheral neuropathy being the most concerning adverse events. Children tend to suffer fewer LZD related adverse effects than adults, but peripheral neuropathy may be difficult to detect and can be irreversible. In children, the most common adverse effects are gastrointestinal disturbance (diarrhea, 9%; vomiting, 4%), which rarely require alteration or discontinuation of the drug. Children receiving LZD should have close monitoring with full blood counts every 2 weeks for the first 2 months and then monthly, with evaluation for neuropathy at each visit and a low threshold for interruption or discontinuation [45]. No adjustment is recommended because of renal impairment or mild-to-moderate hepatic impairment. However, the use of LZD has not been adequately evaluated in the case of severe hepatic impairment (Child Pugh Class C). TDZ data in children are very limited and no evidence-based recommendations can be conducted. 

#### 3.3.8. Other Oxazolidinediones

Sutezolid is a new generation of oxazolidinedione not yet approved by the FDA. In a mice model of TB, it showed an increased bacterial killing and a reduced relapse rate when combined with BDQ and PTM [120]. Another study showed that sutezolid and delpazolid, yet another new oxazolidinone, have a better in vitro activity against *M. abscessus* and *M. fortuitum* [121]. Currently, dose finding studies and combination studies are ongoing (NCT03959566/NCT031993139). Information about delpazolid performance against MTB is lacking.

### 3.4. Fluoroquinolones: Levofloxacin and Moxifloxacin

Fluoroquinolones have in vitro activity against MTB and also have a good penetration into macrophages, which is a particularly important property in view of the ability of mycobacteria to survive and multiply within these phagocytic cells [122]. A new generation of fluoroquinolones (levofloxacin and moxifloxacin) have shown greater activity against MTB than ciprofloxacin and ofloxacin [123]. Moreover, they are dosed once daily, which facilitates treatment adherence. Current guidelines recommend including a new generation fluoroquinolone in all MDR TB regimens unless contraindicated [15].

#### 3.4.1. Mechanism of Action

Fluoroquinolones have a bactericidal effect by inhibiting mycobacterial DNA gyrase, which prevents bacterial DNA from unwinding and replicating [124].

#### 3.4.2. Resistances

Resistance to fluoroquinolones occurs mainly as a result of point mutations within the quinolone resistance determining region (QRDR) in DNA gyrase A (*GyrA*) and Gyrase B (*GyrB*) genes [125]. The levels of resistance are associated with specific mutations within the QRDR of *GyrA*: mutations in subunit A confer high-level resistance, whereas those in subunit B confer low-level resistance [51]. Moreover, different mutations in DNA *GyrA* may confer resistance to both levofloxacin and moxifloxacin, while other mutations confer resistance against levofloxacin, but susceptibility to moxifloxacin is still plausible [126]. 

#### 3.4.3. Posology

The recommended standard dose of moxifloxacin is one 400 mg film-coated tablet once daily, although higher doses of moxifloxacin up to 800 mg daily have also been used with good results and better rates of sputum culture conversion and survival than conventional dose in patients with meningitis TB, as they increase the drug concentration in the affected tissues [127,128,129]. 

Doses between 750 mg and 1.5 g daily of levofloxacin have been used, depending on the patient’s weight [15].

#### 3.4.4. Efficacy

Moxifloxacin has potent in vitro activity against MTB, and its killing activity is like that of INH [130,131]. The favorable pharmacokinetic profile of the moxifloxacin makes it an attractive drug to be used in TB, since it has excellent absorption, bioavailability, and intracellular activities. Moxifloxacin has shown promising results in combination with other anti-TB drugs (both first-line drugs and new drugs). Furthermore, moxifloxacin is considered the main drug to achieve a shortened treatment for TB [132]. Fluoroquinolones constitutes the backbone for treating MDR TB, and the WHO recommend their use in all patients with a MDR TB infection [15]. These recommendations are mainly based in the results of a recent individual data patient meta-analysis in which the use of levofloxacin or moxifloxacin was related with the successful treatment of patients with MDR TB [35]. The choice of levofloxacin or moxifloxacin for treatment of patients with MDR TB may not affect sputum culture conversion at 3 months of treatment [133]. However, in the most recent trials evaluating the efficacy of all-oral and short regimens to treat MDR TB, moxifloxacin has been the fluoroquinolone of choice [20,36,128].

#### 3.4.5. Adverse Effects

The most relevant adverse events related with fluoroquinolones are musculoskeletal disorders and QTc prolongation. Tendinitis may appear early or late after the initiation of fluoroquinolones, appearing even after months of discontinuation the treatment. The risk of tendinitis and tendon rupture is increased in older patients, patients with renal impairment, patients with solid organ transplants, and those treated concurrently with corticosteroids. Regarding QTc prolongation, both moxifloxacin and levofloxacin may cause it. However, moxifloxacin has a small higher risk than levofloxacin in some reviews [134]. Cautious is advised when administering fluoroquinolone with other QTc-prolonging drugs, and close monitoring with EKG is recommended. 

In 2018, the FDA warned about the increased risk of aortic aneurysm or dissection associated with the use of fluoroquinolone based on four epidemiological studies [135,136,137,138]. Since then, the use of fluoroquinolones in patients with aortic aneurysm or with other risk factors predisposing for aortic aneurysm is recommended only after careful benefit–risk assessment. 

Although infrequently, cases of fulminant hepatitis potentially leading to liver failure (including fatal cases) have been reported with moxifloxacin. Quinolones have been also related to sensory or sensorimotor polyneuropathy. Hence, quinolones should be used with caution in patients with central nervous system (CNS) disorders or in the presence of risk factors that predispose to seizures. 

Quinolones cause photosensitivity reactions. Among quinolones, moxifloxacin has a lower risk to induce photosensitivity. Nevertheless, patients should be advised to avoid exposure to either UV irradiation or extensive and/or strong sunlight during treatment with quinolones [139].

#### 3.4.6. Interactions

Quinolone absorption is markedly reduced with antacids containing aluminum, magnesium, and/or calcium and therapeutic failure may result [140]. Fluoroquinolones do not have clinically significant interaction with antiretroviral drugs [141]. In most studies, moxifloxacin has been administered at a dose of 400 mg per day; however, RIF decreases moxifloxacin plasma levels, leading to a 30% reduction in the AUC24 [142,143], although the clinical significance of this reduction is unknown [142,143,144].

#### 3.4.7. Children

Levofloxacin is contraindicated in growing children and adolescents because of theoretical effect on growing cartilage, but it is still a core agent in the treatment of children with MDR TB. Therefore, it may be considered in cases where there are no reasonable alternatives due to the fact of multidrug-resistant pathogens [145]. 

Dosage of 15–20 mg/kg once daily is recommended (upper daily dose 1–1.5 g) for children under 15 years (orally or intravenous) [15].

Dosing for renal impairment is recommended as follows:

1. GFR ≥ 30 mL/min/1.73 m^2^: No adjustment necessary;
2. GFR 10–29 mL/min/1.73 m^2^: 5–10 mg/kg/dose every 24 h;
3. GFR < 10 mL/min/1.73 m^2^: 5–10 mg/kg/dose every 48 h;
4. Intermittent hemodialysis: 5–10 mg/kg/dose every 48 h; not removed by hemodialysis; supplemental levofloxacin doses are not required;
5. Peritoneal dialysis (PD): 5–10 mg/kg/dose every 48 h; not removed by peritoneal dialysis. Supplemental levofloxacin doses are not required;
6. Continuous renal replacement therapy (CRRT): 10 mg/kg/dose every 24 h.

In case of hepatic impairment, there are no dosage adjustments provided in the manufacturer’s labeling; however, dosage adjustment is unlikely to be necessary due to the limited hepatic metabolism of levofloxacin. 

Apart from the effect on growing cartilage, other musculoskeletal adverse events like tendonitis may occur. Gastrointestinal tract disturbances, cardiac disturbances, peripheral neuropathy, rash, headache, restlessness, and confusion are other reported adverse effects. 

There are limited data available for moxifloxacin. The recommended dosage is 10–15 mg/kg orally or IV (upper daily dose: 400 mg) [15,18]. Safety concerns for moxifloxacin are similar to levofloxacin. Based on experience in adult patients, no dosage adjustment is necessary in the case of renal or hepatic impairment.

### 3.5. Clofazimine (CFZ)

CFZ is a lipophilic riminophenazine antibiotic with both antimycobacterial and anti-inflammatory activities that have been mainly used in the management of leprosy [146].

#### 3.5.1. Mechanism of Action

The mechanism of action of CFZ against mycobacteria is not completely understood. An in vitro study showed that CFZ releases lysophospholipids, which is toxic to mycobacteria [147]. Another study showed that CFZ induces apoptosis in the human macrophage with fragmentation of the DNA [148]. Other studies have reported a reduction in the availability of ATP caused by CFZ [149].

#### 3.5.2. Resistance

As mentioned before, both BDQ and CFZ share the same efflux pump system, and cross-resistance to both drugs have already been reported [29]. 

The main mechanisms of resistance to CFZ are: 

Mutations in the locus *rv0678*, which encodes a transcriptional repressor for the efflux pump *MmpL5*, leading to an upregulation of the multi-substrate efflux pump involved in drug resistance. It also confers cross-resistance to BDQ; 

Mutations in the gene *pepQ* (Rv2535c) linked with low-level resistance to BDQ as well;

Mutation in rv1979c, which is believed to be related with transmembrane transporter with permease activity [150].

#### 3.5.3. Posology

According to the WHO’s MDR TB guidelines, CFZ is administered in a 100 mg dose once daily and it has better absorption if taken with a high-fat and -protein meal [146]. High-dose CFZ (equal or superior to 200 mg daily) has been used in clinical practice in patients with non-responsive MDR or XDR TB with good results when patients were over 50 kg, otherwise adverse events comprise a successful outcome [151]. PK/PD studies of CFZ also recommend weight-based dosing [152]. In fact, the 2008 WHO guidelines recommended a CFZ dose of 200–300 mg for the first months of treatment in patients with MDR TB [153].

#### 3.5.4. Efficacy

The potential role of CFZ for improving the treatment outcome of patients with MDR TB was observed in a clinical trial conducted in Bangladesh [5]. In this trial, a relapse-free cure of 87.9% patients was observed after a 4 month intensive phase with prothionamide, kanamycin, high-dose INH, gatifloxacin, CFZ, ethambutol, and pyrazinamide followed by 5 months of gatifloxacin, CFZ, ethambutol, and pyrazinamide. In 2015, the addition of CFZ to individually based chemotherapy regimens were observed to accelerate sputum culture conversion and improve treatment success rates [13]. Additionally, a randomized multicenter study carried out in China observed that the proportion of patients with favorable outcomes was significantly higher in patients receiving CFZ plus a standard regimen compared with patients receiving the standard treatment without CFZ [154]. In a recent clinical trial, a 12 months regimen containing CFZ was not inferior to an 18 month standardized regimen without CFZ [155].

Currently, CFZ is included in the group B medicines recommended for use in MDR TB cases, and it is one of the drugs included in the shorter all-oral BDQ-containing regimens recommended by the last WHO guidelines [15].

#### 3.5.5. Adverse Events

Main adverse event related with the use of CFZ is skin pigmentation and gastrointestinal intolerance [156,157,158]. Most of the adverse effects related with the use of CFZ are mild and do not require treatment interruption, although some reports have documented severe abdominal pain, presumably due to the accumulation of CFZ crystals in the intestinal mucosa [159]. Administration of higher doses of CFZ in underweight patient is associated with a 2.57-fold increase in the likelihood of experiencing an adverse event [151].

#### 3.5.6. Interactions

CFZ has reversible inhibitory effects on P450 in in vitro studies. CFZ was predicted to be a moderate-to-strong CYP3A4/5 inhibitor using both static and dynamic modeling approaches. However, the clinical implication of this interaction remains unknown [160].

#### 3.5.7. Children

The pharmacokinetics of CFZ in children has not been studied and to date there are no planned trials to evaluate this Group B drug. However, the current WHO recommendation is a dose of 2–5 mg/kg per day (maximum dose: 100 mg daily) for children [15]. Owing to the long half-life of CFZ, lower doses on alternate days could be considered in younger children, as capsules, which are currently the only available presentation, cannot be split [18].

CFZ should be given for the entire duration of therapy if it is tolerated. Rates of reversible red-grey skin discoloration are high (~90%) and can lead to social stigmatization but rarely result in discontinuation with adequate counseling. In the absence of clear safety data in children, a monthly ECG if CFZ is used with other QTc-prolonging drugs is recommended [45]. Frequent gastrointestinal toxicity has also been described [161].

### 3.6. Aminoglycosides and Capreomycin

The role of aminoglycosides (AGs) in the treatment of TB is a history of rise and fall. Streptomycin was the first drug that was successful against TB and was part of the standard retreatment scheme (category II) recommended by the WHO until 2017 [162,163]. Until very recently, AGs were part of the backbone of any MDR TB regimen together with fluoroquinolones and AG resistance was part of the definition of XDR-TB until it was updated in October 2020 [17]. The other AGs commonly used in TB treatment are amikacin and kanamycin. 

A common misunderstanding is considering capreomycin as another aminoglycoside, but it is a cyclic polypeptide (like viomycin) [164]. However, due to the similar routes of administration, mechanisms of action and toxicity profiles, they are traditionally grouped together as injectable drugs for TB.

Despite AGs not currently being a part of the recommended regimen for MDR TB, they are still an alternative drug with potent bactericidal activity for the use in personalized treatment oof patients with non-responsive MDR or XDR TB. 

Currently, only amikacin and streptomycin are recommended for the treatment with longer regimen for patients with MDR TB, since the use of kanamycin and capreomycin is associated with a poorer outcome [15].

#### 3.6.1. Mechanism of Action

AGs bind to the ribosomal 16S subunit hence inhibiting protein synthesis. Not all AGs cover the same rRNA bases in the active site. For example, neomycin, paromomycin, gentamicin, and kanamycin bind in a combination of bases (A1408 and G1494), and amikacin in a different combination, thus explaining the incomplete cross-resistance in some situations [165]. AGs also affect the integrity of the bacterial cell membrane by disrupting magnesium bridges between lipopolysaccharides [166]. Capreomycin acts at the interface area that mediates bridging between the 30S and 50S ribosome subunits, blocking the ribosome function [167].

#### 3.6.2. Mechanism of Resistance

Bacteria develop resistance to AG using enzymatic deactivation, changes in transport mechanisms, alterations in the 30S ribosomal subunit, and modifications in the methylation status of the rRNA [168]. This span of mechanisms partly explains the different patterns of resistance to AG, and the challenges for explaining the phenotypic resistance with genetic analysis.

In MTB, there are some mutations that are associated with resistance to AG, mainly in the rRNA binding site. The A1401G and G1484T mutations of the *rrs* encoding the 16S rRNA are associated with resistance to amikacin, kanamycin, and capreomycin. In contrast, the C1402T mutation is associated with capreomycin resistance, but not to amikacin or kanamycin resistance. Mutations in the *eis* gene promoter region are related to kanamycin resistance but can be found in either capreomycin-susceptible or capreomycin-resistant strains. Mutations in tlyA methyltransferase cause loss of methylation in C1409 of the 16S rRNA and C1920 of the 23S rRNA, conferring resistance to capreomycin but not to other AGs [167,169]. 

#### 3.6.3. Posology

AGs are not absorbed through the gastrointestinal tract. Therefore, their use implies intramuscular or intravenous injections. They are weakly bound to proteins, so high concentrations of free drug can be found in plasma and interstitial fluid. However, their penetration into the CNS and through other body membranes is poor, except for the renal tubules, the middle ear (which explains main toxicity targets of AGs), and synovia [170]. In Gram-negative bacteria, AGs enter the periplasmic space by passive diffusion through porin channels in the outer membrane, and the cytoplasm by active transport depends on the electron transport chain [171,172]. 

In TB, AGs and capreomycin are administered once daily by intravenous or intramuscular injection.

Amikacin is given at 15–20 mg/kg/day, kanamycin’s optimal dose is 15 mg/kg/day, and capreomycin’s recommended dose is approximately 15–20 mg/kg. In the latter case, however, as vials have about 1 g of capreomycin, it is common practice to use this dose for most patients [153]. 

The recommendation has been to maintain injectable drugs for a minimum of 4 months or at least until the patient suffers renal or ototoxicity or adverse events related to the route of administration. The continuation phase should avoid AGs when possible [5]. When the intramuscular route is used, rotation of injection sites is advised to reduce discomfort. Other posology including three day per week administration or discontinuation during weekends has been explored with promising results. However, from a PK perspective, daily administration is preferred and decrease the risk of resistance amplification during treatment.

Therapeutic drug monitoring is not usually performed for injectable drugs in TB, although evidence of drug adjustment based on AG levels exist in bacterial infection. If there are no other alternatives for a patient with a considerable risk of toxicity than to use AGs, therapeutic drug monitoring should be performed to minimize such risk, although evidence supporting specific goals are scarce and mainly based on small case series [173].

#### 3.6.4. Efficacy

For many years, injectable drugs, such as amikacin, kanamycin, and capreomycin, were considered an essential part of the treatment of MDR TB [153]. Additionally, injectable drugs were part of the first short duration treatment for MDR TB, based on the results from the short Bangladesh regimen, later confirmed in the STREAM trial [4,5,128].

However, in a recent individual patient data meta-analysis, including 12,030 cases, receiving amikacin was associated with increased treatment success (adjusted odds ratio, aOR 2.0, 95% CI 1.5–2.6), although risk of death was similar (aOR 1.0, 95% CI 0.8–1.2) compared to patients that did not receive amikacin. The analysis was adjusted by strain susceptibility, smear positivity, and pulmonary cavitation. Kanamycin and capreomycin were associated with less treatment success (aOR 0.5, 95% CI 0.4–0.6 and 0.8, 95% CI 0.6–1.1, respectively) and capreomycin with an increased mortality (aOR 1.1, 95% CI 0.9–1.2 and 1.4, 95% CI 1.1–1.7, respectively) [35]. This evidence, combined with their safety profile and route of administration has made it that so only amikacin holds a place in the treatment of MDR TB when no other oral or safer drugs are available [15,19,20].

In addition, infection-site conditions affect the activity of AG. Since these are basic substances conformed by a chain of sugars with glycosidic bonds to a cyclic alcohol with amino radicals, acid pH and divalent cations deactivate these molecules. Therefore, their efficacy is jeopardized in abscesses, bronchial secretions, and necrotic tissue. Furthermore, as their transport into the cytoplasm is an active process, AGs are less effective in anaerobic environments where the electron transport chain is not active [166].

#### 3.6.5. Adverse Events

Both AGs and capreomycin have a significant risk of renal toxicity and ototoxicity. In general, about 5–25% of the patients receiving AGs will suffer a deterioration of serum creatinine levels which is mild to moderate and transitory in most cases [168]. The risk of nephrotoxicity is reduced but not eliminated when AGs are administered in a single daily dose [174].

Ototoxicity can take the form of auditory and vestibular toxicity. Hearing loss is independent from renal toxicity and is often irreversible and progresses from higher to lower frequencies. Therefore, when a patient refers losing conversation hearing, the damage is quite advanced. Vestibular toxicity (vertigo, gait instability) is parallel to auditory toxicity but is often reversible [175].

In the Bangladesh trial, 19 out of 427 participants (4.4%) had hearing loss [5]. In the STREAM trial, 29 out of the 423 participants (7%) had ototoxicity including hearing loss and vertigo, and 14 (3%) had renal adverse events without significant differences between the long- and the short-duration arms, suggesting that most adverse events happen during the first months of treatment [128].

If toxicity in the form of either renal function or hearing loss happens, and there are alternative drugs available, injectable drugs should be discontinued.

The guidelines recommend at least monthly monitoring of the renal function and audiometry, but this should be personalized according to each patient’s basal risk for toxicity and resources availability.

Other more common but less severe adverse events include skin rash, nausea, and vomiting. Dizziness, headache, fever, anemia, eosinophilia, hypomagnesemia, polyneuropathy, muscle tremors, joint pain, low blood pressure, and itching have been described with the same frequency as kidney impairment and hearing [170].

#### 3.6.6. Interactions

AGs interact with loop diuretics such as furosemide and torasemide. The two classes of drugs can act synergistically causing hearing loss, especially when the renal function is impaired. Nephrotoxicity may be cumulative when AGs are administered together with other nephrotoxic drugs such as amphotericin B or cidofovir. Inactivation of the AG by beta-lactams is described in the literature, although its clinical significance is uncertain [170]. In addition, AG can cause a neuromuscular blockade when co-administered with non-depolarizing muscle relaxants, and their concomitant use should be avoided. Opioids in combination with AGs can increase their effect as respiratory depressants [176]. 

#### 3.6.7. Children

The avoidance of an injectable-containing regimen is particularly desirable in children [15]. However, if there is need to use aminoglycosides, the daily dose for amikacin is 15–20 mg/kg IM or IV adjusted according to serum concentrations (maximum daily dose: 1 g) and for capreomycin is 15–30 mg/kg IM (maximum daily dose: 1 g). The main serious adverse effects are ototoxicity, vestibular toxicity, nephrotoxicity, electrolyte disturbances, and local pain with IM injections for both.

### 3.7. Ethionamide and Prothionamide

Both ethionamide and prothionamide are thionamide drugs that have been commonly used in the MDR TB treatment.

#### 3.7.1. Mechanism of Action

Like INH, ethionamide and prothionamide are pro-drugs that need activation by monooxygenase *EthA* [177,178]. After activation, thionamides inhibit the mycobacterial synthesis of mycolic acid through the inhibition of the *InhA* enzyme [179].

#### 3.7.2. Resistances

Resistances to thionamides are associated to the following mutations: 

Mutations in the *inhA* promoter region, which results in co-resistance to INH [180];

Mutations in the *ethA* gene encoding the activation of the drug, thus preventing drug activation [181]. Isoniazid susceptibility is not affected;

Mutations in the *mshA* and *ndh* genes. *MshA* encodes an enzyme that promotes the activation of ethionamide [182]. Mutations in the *ndh* gene results in increased intracellular NADH concentration, competitively inhibiting the binding of INH–NAD and ETH–NAD therefore leading to co-resistance of INH and ETH [183].

#### 3.7.3. Posology

Ethionamide is administered at a dose of 15–20 mg/kg/day, usually as 2–3 divided doses. Food and antacids have no effect on the absorption [184,185]. 

Prothionamide’s recommended dose is 15–20 mg/kg/day, and the maximum dose per day should not exceed 1 g. The dosage can be taken once daily or split in two to three doses if not tolerated. 

#### 3.7.4. Efficacy

Several studies published before 1970 showed the efficacy of ethionamide in sputum conversion in patients with TB when accompanied by one or two other drugs [186,187,188]. However, in a meta-analysis published in 2018, the use of ethionamide did not show any benefit in patients with susceptible isolates [35].

The 2019 WHO consolidated guidelines do not recommend the use of thionamides, except when more effective agents (e.g., BDQ, LZD, and CFZ) cannot be used [15]. They should be used in combination with other effective drugs in patients with MDR TB.

#### 3.7.5. Adverse Events

Main adverse effect is gastrointestinal intolerance, which is dose-related and often improves after two to four weeks of therapy [189]. Some studies have shown that prothionamide is better tolerated than ethionamide [190]. When the drug is taken with meals it usually improves gastrointestinal intolerance. Hepatotoxicity may also occur, although severe hepatotoxicity is rare [191]. Ethionamide has also been related with hypothyroidism when used for long periods, which is reversible after drug cessation [192,193]. CNS toxicity (psychosis, seizures, and behavioral disorders) has also been associated with thioamide use [194,195,196]. Gynecomastia is another possible adverse effect [197,198,199]. 

A systematic review comparing the efficacy and tolerability of both thionamides concluded that prothionamide may be slightly more effective and better tolerated that ethionamide [200]. Ethionamide was one of the drugs related to more adverse events in a prospective study evaluating the frequency and severity of adverse events related to anti-TB drugs [201].

#### 3.7.6. Interactions

No relevant interactions have been described.

### 3.8. Cycloserine and Terizidone

Cycloserine is a bacteriostatic drug and is part of the group B of the grouping medicines recommended for use in longer MDR TB regimens. Terizidone is a structural analogue of cycloserine. Cycloserine and terizidone are considered interchangeable for the treatment of MDR TB.

#### 3.8.1. Mechanism of Action

Cycloserine is a cyclic analogue of d-alanine and blocks the formation of the bacterial cell wall by targeting the alanine racemase and D-alanine ligase [202].

#### 3.8.2. Posology

The dose of cycloserine is 10–15 mg/kg orally, given in divided doses 1–2 times a day. The maximum recommended dose is 1000 mg/day

#### 3.8.3. Efficacy

Cycloserine has been used since 1950 for the treatment of TB [203]. An observational prospective study of patients with MDR TB treated with a standard background regimen plus cycloserine, terizidone, or ethambutol showed that cycloserine achieved higher culture conversion rates than terizidone [204]. The use of cycloserine should follow DST results or if the prevalence of resistance in the community is less than 10%. In an individual patient data meta-analysis for longer MDR TB regimens, the addition to cycloserine or terizidone was associated with an increase likelihood of treatment success vs. treatment failure or relapse and with less risk of death (aOR 0.6, 95% CL 0.4–0.9 and 0.6, 95% CL 0.5–0.8, respectively) [35].

#### 3.8.4. Adverse Events

Main adverse events associated with cycloserine are psychiatric disorders and CNS toxicity, including seizure, depression, psychosis, and suicidal ideation. Other adverse effects include peripheral neuropathy and skin changes. Skin problems include lichenoid eruptions and Stevens–Johnson syndrome [205]. Terizidone has been related with less adverse events compared to cycloserine [204]. A careful assessment of patient medical history is recommended to decide the drug of choice of group B for longer MDT TB regimen (CFZ and clycloserine/terizidone).

#### 3.8.5. Resistances

The mechanisms of cycloserine resistance are complex and involve genes participating in lipid metabolism, stress response, and transport system [206]. Neither cycloserine nor terizidone present cross-resistance with other TB drugs [207].

#### 3.8.6. Interactions

The combination with other medications affecting the CNS can increase the risk of seizures and other drug-induced psychiatric diseases. No other relevant interactions have been described [201].

#### 3.8.7. Children

Although efficacy and safety of cycloserine in children has not been well established, it has been widely used for MDR TB treatment in children. 

The recommended dose is 15–20 mg/kg orally in one dose or two divided doses (maximum daily dose: 1 g) [15]. When available, serum drug monitoring is advised to establish optimal dosing. The recommended peak (two to four hours post-dose) level should not be higher than 30 mcg/mL [15].

Psychosis, personality changes, seizures, and rashes have been described as adverse effects. Treatment should be permanently discontinued if neurological or psychiatric events occur [149]. 

## 4. Discussion

In spite of the current revolution in MDR TB management strategies, including the diagnosis of molecular resistances and the tailored all-oral short regimens founded in an ever-growing body of evidence, there are several caveats that still make MDR TB a public health concern and an unsolved medical need.

First, as it can be inferred from the manuscript, there are several possible combinations of drugs and durations in the clinical guidelines with no clear preference for any of them. Because of its complexity, the composition and follow-up adaptations of the treatment regimens often needs a team of experts (“TB Consilium”), which is not available everywhere and thus makes the idea of a decentralized care for MDR TB patients quite a challenge. Additionally, some of the recommended regimens and durations were randomly selected, and optimizations should carefully be adopted based on the characteristics of the patients. For example, in the current guidelines all patients are recommended the same duration regardless the burden of diseases and the early response to the treatment; and the short MDR TB regimen includes drugs that have less in vitro and in vivo efficacy than drugs from the A group. Other aspects are the use of drugs not included in group A in the short MDR TB regimens or the use of high-dose INH even in the presence of high-level resistance. Additionally, BDQ and DLM have been used for longer periods than the approved 6 months, and based on their efficacy data and safety profile, recommendations should extend their use beyond 6 months. All these considerations highlight the need for a tailored treatment for the patients with MDR TB and the need to gather real world data to answer these burning questions. Hopefully, this article can help clinicians to understand the peculiarities of the main compounds that are in use for MDR TB. Such guidance should not be understood as a fixed rule, but rather general concepts must guide an adaptive way of thinking. 

This complexity offers some opportunities. As new drugs and regimens need supporting evidence, the research needed to obtain such evidence is the perfect place to involve relevant stakeholders together [208]. Community and patients must work with clinicians, researchers, industry representatives, and regulators to build strong networks that accelerate drug and regimen development in a way that is relevant and accepted by its final users [209]. As for many other conditions, TB research can be the opportunity to build and strengthen research and clinical capacities in low-income settings, increasing the quality standards of healthcare for those that are most affected by this disease [210].

The emergence of new, highly effective regimens may open a new way around RIF and INH resistance. Within a few years, we may see the first pan-TB regimen trials, aiming for a unique solution for both MDR and Ds-TB [211]. This will have the advantage of an easier operational implementation [212]. However, we must recall that the classic exposure was once a “pan-TB” regimen and that new first-line treatment may lead to new profiles of TB resistances [213]. 

Development and implementation require resources, both human and material. In many settings, healthcare personnel are so overwhelmed by daily duties that changes are introduced at a very slow pace [214,215]. The apparent increase in MDR TB incidence that has been reported after the implementation of Xpert MDR^®^ is due to the detection of more cases that shortly before were treated with first-line drugs empirically, hindering the control of the TB pandemic and farming resistant strains [216,217]. New diagnostic tests in TB with an evidence-based benefit in TB management should be rapidly adopted, ensuring that resources and capacity building are ready since they are as important as having the diagnostic test equipment and fungibles. Drug availability is another concern. Global efforts to ensure drug availability in resource-limited settings help with this problem, but sometimes generate disparity between sites. For example, whilst BDQ is widely available in resource-limited settings, [218], it is not easily available in Europe. A global effort to ensure TB drug supplies should be done and lead coordinately by the WHO with rapid roll out of new drugs after a careful evaluation of the risk-benefit trade-off. 

Patients with TB often suffer isolation and stigma, but this is by far more patent in patients with MDR TB. Some of the treatments have visible signs of their use (e.g., skin depigmentation with CFZ) [219,220,221]. This causes havoc amongst those with a weak social and familiar support, which may result in poor drug adherence leading to resistance amplification and community expansion. As new drugs will be marketed in the coming years, old implementation problems need to be approached with innovative solutions. Current guidelines recommend that TB care is delivered by multidisciplinary teams with social workers and psychologists encompassing all aspects of the patient with TB.

Finally, in many settings, a subacute disease with social implications such as TB has economic consequences for the household as a unit. One of the EndTB pillars is the elimination of catastrophic costs for those who suffer from TB. These costs are defined as both direct (what the family needs to pay for) and indirect (how much the household has not earned due to the disease) [130]. According to the WHO 2020 global report, about 45% of households suffer catastrophic costs, but the proportion increases to near 80% for the MDR TB subgroup [1]. Long, complex, and toxic treatments increase the costs for the patients and their families and thus make it difficult for them to complete the entire treatment. Again, to fight TB we need more than novel and effective drugs, we need to cover of the problems that the patient with TB suffering, including, among others, stigma, malnutrition, financial difficulties, and substance abuse.

This was a narrative, non-systematic review. Therefore, it must not be considered as a clinical guideline or as a showcase for all the current evidence about novel treatments. Second, our recommendations are indefectibly affected by our context. Some of the perspectives presented in this article must be adapted to the reality of each country’s healthcare system. 

## 5. Conclusions

During the last several years significant changes have occurred in MDR TB treatment. There are new and repurposed drugs with good bactericidal and sterilizing activity and several all-oral short regimens have appeared showing promising results. The availability of rapid and sensitive molecular tools to diagnose drug resistance that compose these new regimens will be essential to treat patients with the best combination and avoid resistance emergence. Patients with MDR TB benefit from close monitoring and expert broad knowledge in TB drug follow up, since a drug regimen is usually modified during treatment due to the toxicity or phenotypic drug sensitivity test.

## Figures and Tables

**Table 2:** Summary of drugs for MDR-TB.

WHO Class	Drug	Short Name	Effect	Dose	Interactions	AE Monitoring
A	Bedaquiline	BDQ	Bactericidal	Standard: * 400 mg po once daily for 2 weeks 200 mg po three times a week Alternative: 200 mg po once daily for 8 weeks 100 mg po daily Children (max. dose 400/200 mg): 6 mg/kg/day once daily for 2 weeks 3–4 mg/kg thrice weekly	CYP3A4 inhibitors: PI and efavirenz	Long QT: basal, 2 weeks and then monthly ECG
	Linezolid	LZD	Bacteriostatic (in vitro model have shown bactericidal activity)	Standard: 1200 mg once or 600 mg po/iv twice a day Alternative: Adjust to 600 mg po/iv daily after 3–4 w of standard dose. Doses up to 300 mg po/iv daily with effective adjuvant treatment Children (max. dose 600 mg/day): ≥16 kg: 10–12 mg/kg/day po/iv once daily <16 kg: 15 mg/kg/day po/iv once daily	See Table A1 Interaction with drugs that increase serotonin levels. Avoid coadministration with pethidine, tramadol, methadone, or fentanyl	Haematology (myelosuppression): complete blood counts every 2–4 weeks Evaluate for early clinical signs of peripheral neuropathy. There is no clear recommendation for the routine use of EMG.
	Levofloxacin	LFX	Bactericidal	Standard: 750 mg po/iv once daily Alternative: 1500 mg po/iv once daily Children (max. dose 1–1.5 g/day): 15–20 mg/kg po/iv once daily	Caffeine: increases systemic effect, more frequent with ciprofloxacin, less with LFX; may lower seizure threshold Avoid coadministration with drugs that can prolong the QTc.	Long QT: like BDQ Tendinitis signs must be checked at every visit. Patients should be advised about the risk of tendon rupture Precaution in patients with aortic aneurysms, but no monitoring is currently recommended
	Moxifloxacin	MFX	Bactericidal	Standard: 400 mg po once daily Alternative: 600–800 mg po once daily (especially with coadminister with drugs that reduce AUC0-24) Children (max. dose 400 mg/day): 10–15 mg/kg po/iv once daily	Absorption markedly reduced with antacids based on magnesium, aluminium, and calcium Rifampicin may reduce MFX exposure in 30%	Long QT: like BDQ Tendinitis signs must be checked at every visit. Patients should be advised about the risk of tendon rupture Precaution in patients with aortic aneurysms, but no monitoring is currently recommended
B	Clofazimine	CFZ	Bactericidal	Standard: † 100 mg po once daily Alternative: ≥200 mg po once daily (patients with >50 kg) Children (max. dose 100 mg/day): 2–5 mg/kg po once daily	Moderate CYP3A4/5 inhibitor, but clinical significance is uncertain	Long QT monitoring if used with other drugs with potential cardiotoxicity Skin pigmentation is frequent, but does not imply treatment discontinuation and is often reversible
	Cycloserine/terizidone	CYS/TRZ	Bacteriostatic	Standard (max. dose 1000 mg/day): 10–15 mg/kg po in one or two doses daily Children (max. dose 1000 mg/day): 15–20 mg/kg po in one or two doses daily	May lower seizure threshold when administered with other pro-epileptogenic drugs	Close monitoring for neuropsychiatric AE TDM recommended for children to avoid toxicity
C	Delamanid	DLM	Bactericidal and sterilizing	Standard: * † >50 kg: 100 mg po twice a day 30–50 kg: 50 mg po twice a day Children (max. dose 200 mg/day): <3 years: no clear dosage (3–4 mg/kg/day?), may need higher doses than 3–5 years 3–5 years: 25 mg po twice daily 6–11 years: 50 g po twice daily 12–17 years: 100 mg po twice daily	No relevant interactions	Long QT: like BDQ
	Amikacin	AMK	Bactericidal	Standard: 15–20 mg/kg iv/im once daily Alternative: 15–20 mg/kg iv/im 2–3 times a week Children (max. dose 1000 mg/day) 15–20 mg/kg iv/im once daily (adjust according to serum concentrations)	Toxicity can be cumulative. Ototoxicity: loop diuretics (i.e. torsemide) Nephrotoxicity: amphotericin, cidofovir Betalactams may inactivate AG	Monthly check renal function and audiometry, although this should be tailored to baseline risk TDM can be performed if elevated risk of toxicity and no alternative to injectable drugs
	Ethionamide/prothionamide	ETH/PTH	Bacteriostatic	Standard (max. dose 1000 mg/day): 15–20 mg/kg po in two or three doses daily Children (max. dose 1000 mg/day): 15 to 20 mg/kg po in 2 or 3 divided doses	No relevant interactions have been described	Electrolyte and renal function should be monitored if severe gastrointestinal AE develop Monitor thyroid function periodically in patients at risk
Other	Kanamycin	KAN	Bactericidal	Standard: 15 mg/kg iv/im once daily Alternative: 15 mg/kg iv/im 2–3 times a week	Toxicity can be cumulative. Ototoxicity: loop diuretics (i.e. torsemide) Nephrotoxicity: amphotericin, cidofovir Betalactams may inactivate AG	Monthly check renal function and audiometry, although this should be tailored to baseline risk TDM can be performed if elevated risk of toxicity and no alternative to injectable drugs
	Capreomycin	CAP	Bactericidal	Standard: 15–20 mg/kg iv/im once daily Alternative: 15–20 mg/kg iv/im 2–3 times a week Children (max. dose 1000 mg/day): 15 to 30 mg/kg im once daily	Toxicity can be cumulative. Ototoxicity: loop diuretics (i.e. torsemide) Nephrotoxicity: amphotericin, cidofovir Betalactams may inactivate AG Increases neuromuscular blockade of non-depolarizing muscle relaxants	Monthly check renal function and audiometry, although this should be tailored to baseline risk TDM can be performed if elevated risk of toxicity and no alternative to injectable drugs
	Pretomanide	PTM	Bactericidal and sterilizing	Standard: * † 200 mg once daily Children: Scarce data available, delamanid recommended as alternative	Rifampicin and efavirenz may reduce PTM levels, less interference with lopinavir/ritonavir	Long QT: like BDQ
	Tedizolid	TZD	Bacteriostatic	Standard: 200 mg po/iv daily Children: Scarce data available, linezolid recommended as alternative	See Table A1 Coadministration with drugs that increase serotonin level may induce a serotonin syndrome	Although side effects appear to be less frequent in some studies, similar haematological and neurological monitoring as linezolid is recommended.

BDQ and DLM use is approved for 24 weeks, but there is increasing evidence with longer durations. * DLM and PTM, CFZ are better absorbed when administered with food, † DLM and PTM, CFZ are better absorbed when administered with food.

## Appendix A

**Table A1:** List of drugs with Serotonergic Effects in Central Nervous System.

Increase serotonin formation	Tryptophan, oxytriptan
Increases release of serotonin	Amphetamines and derivatives MDMA (ecstasy) Cocaine Mirtazapine
Impairs serotonin reuptake	MDMA (ecstasy) Cocaine Meperidine Tramadol Pentazocine Dextromethorphan Citalopram Selective Serotonin Reuptake Inhibitors (citalopram, fluoxetine, paroxetine, sertraline) Serotonin-Norepinephrine Reuptake Inhibitors (desvenlafaxine, duloxetine, venlafaxine) Sibutramine Bupropion Serotonin modulators (trazodone, vortioxetine) Cyclic antidepressants (amitriptyline, desipramine, nortriptyline) St John’s Wort 5-HT3 receptor antagonists (dolasetron, granisetron, ondansetron) Cyclobenzaprine Methylphenidate
Inhibits serotonin metabolism by inhibition of MAO	MAO-A inhibitors (methylene blue) MAO-B inhibitors (rasagiline, selegiline)
Direct serotonin receptor agonist	Buspirone Triptans (rizatriptan, sumatriptan) Ergot derivatives (methylergotamine, dihydroergotamine) Fentanyl LSD
Increases sensitivity of postsynaptic serotonin receptor	Lithium

LSD: Lysergic Acid Diethylamide; MAO: Monoamine Oxidase.
